# Exploring the Experiences of Adults With Microtia: A Qualitative Study

**DOI:** 10.1177/1055665620931611

**Published:** 2020-07-09

**Authors:** Claire Hamlet, Diana Harcourt

**Affiliations:** 1 Centre for Appearance Research, University of the West of England, Bristol, United Kingdom

**Keywords:** microtia, hemifacial microsomia, ear reconstruction, psychosocial, quality of life, support

## Abstract

**Objective:**

Microtia is a medically complex condition, with the option of surgery to address hearing and reconstruct the ear. The current study explored adults’ experiences of microtia, with a particular focus on the psychosocial impact and experiences of ear reconstruction. The ultimate aim was to identify areas for support and future research that could improve patient care.

**Design:**

Fifteen adults (12 females) aged between 20 and 62 years took part in semi-structured interviews. Interviews were audio-recorded, transcribed verbatim, and analyzed using inductive thematic analysis.

**Results:**

Three main themes were identified in the data: microtia as an invisible difference, surgery as a welcome opportunity, and living well with microtia. Participants had incorporated microtia into their self-concept and did not report a lasting negative impact on their lives. However, some psychosocial challenges were reported, including anxiety about showing their ears (even after reconstruction), disclosing their diagnosis to romantic partners, surgical decision-making, and feeling unsupported in the work environment.

**Conclusion:**

Individuals with microtia may benefit from psychosocial interventions to increase confidence, access to support for treatment decision-making, and guidance around disclosing microtia to employers.

## Introduction

It is estimated that around 1 in 6000 people in the United Kingdom are born with microtia, a craniofacial condition referring to a malformation or misshape of the external part of the ear. Microtia can range in severity from perfectly formed, smaller than average ears, to ears that are virtually absent (Henderson and Moffat, 2019). Microtia can occur in isolation or as part of other conditions such as hemifacial microsomia and Treacher Collins syndrome (Zim et al., 2017).

Microtia is a complex condition, presenting numerous challenges that require long-term, multidisciplinary care. Microtia often occurs alongside atresia, which may require surgery to restore hearing (eg, with bone-anchored hearing aids). Auricular construction (also known as ear reconstruction) is often recommended to decrease the psychosocial impact of living with a craniofacial difference (Brent, 2002). One surgical option is using rib cartilage to create a 3-D ear framework, typically performed when the child reaches 9 to 10 years old (Cugno and Bulstrode, 2019). Another option is using porous polyethylene implants instead of rib cartilage to create a framework (Yang et al., 2009; Reinisch and Lewin, 2009). An ear prosthesis can also be used to restore form, which is attached using adhesive or bone-anchored titanium implants (Zhang et al., 2019).

Microtia is a highly visible condition due to the prominent location of the ears, although the condition can be concealed using hair or head accessories (eg, hats or headbands). Living with a craniofacial condition can present numerous challenges to psychosocial well-being, including staring, bullying, teasing, and unwanted questions (Rumsey and Harcourt, 2004). Research exploring the psychosocial impact of microtia has primarily been conducted within surgical settings outside of the United Kingdom. For example, psychosocial issues such as depression, anxiety, and social difficulties were found in studies including both children and adults presenting for surgery in China (Jiamei et al., 2008; Li et al., 2010). While quantitative research is useful to test hypotheses, it fails to understand lived experience from speaking directly to those with microtia. Johns et al. (2018) interviewed parents and children with isolated microtia in the United States to understand the early experiences of families. Parents reported stressful experiences of being informed of the diagnosis and experienced multiple negative emotions (eg, guilt, anger, anxiety). Furthermore, their coping was linked to education about etiology, treatment options, normalization, and support from family, health care providers, and support groups. Hamilton et al. (2018) used interviews to explore the medical and psychosocial concerns of 11 adolescents and young people with craniofacial microsomia. They found that adolescents exhibited resilience and described positive personality traits as a result of their condition, such as being more open-minded, less judgmental, and more independent. However, difficulties with bullying and confidence impacted their sense of self.

There appears to be little research focusing on the psychosocial impact of microtia beyond adolescence and outside of surgical settings. While it is possible that the difficulties reported by adults with other craniofacial conditions may present in adults with microtia, this has not yet been explored. Furthermore, ear reconstruction techniques have advanced over the last 20 years or so (Bly et al., 2016), and it is likely that many adults with microtia may not have had reconstruction as a child or made the decision to undergo it later in life. Therefore, adults’ experiences of microtia may differ to those of children and adolescents.

In summary, microtia is a medically complex condition, with the option of surgery to address hearing and reconstruct the ear. There is a lack of research pertaining to the psychosocial impact of microtia, particularly in adults, presenting a need to advance research in this area. The current study explored the experiences of adults with microtia, with a particular focus on the psychosocial impact of the condition, and experiences of ear construction. The ultimate aim was to identify areas for support or future research that could improve patient care.

## Methodology

Ethical approval was obtained from the University of the West of England’s Research Ethics Committee. Recruitment was facilitated by the charity Microtia UK and the Centre for Appearance Research who advertised the study on their social media channels. Inclusion criteria were a diagnosis of microtia, residing in the United Kingdom, aged 18 years or over, and willing to share their experiences. Despite hemifacial microsomia being a condition with additional appearance changes than isolated microtia, those with the condition were not excluded from participating, as the interview schedule (Appendix A), analysis, and reporting focused on experiences of microtia specifically.

Participants were advised they could complete their interview in the way they felt most comfortable (face-to-face, telephone, Skype). However, time and budget constraints meant the geographical location of participants influenced whether it was feasible to offer the option of a face-to-face interview. Consent to participate in the study was obtained on paper for each of the face-to-face interviews and Skype interview and via audio recording for the telephone interviews.

The interview included questions about participants’ experiences of microtia, the psychosocial challenges and opportunities they had encountered, and experiences of decision-making for surgery. An individual with lived experience of microtia provided feedback on the interview schedule before it was finalized. Interviews were semi-structured, so that emerging topics could be explored. All of the interviews were conducted by the first author, who has no personal experience of microtia and/or hearing loss. They were recorded and then transcribed verbatim.

Transcribed interviews were subject to an inductive thematic analysis (Braun and Clarke, 2006), whereby the aim was to generate new themes and concepts, rather than to test existing ones. The transcripts were analyzed by coding extracts to build overall themes using the 6 steps outlined by Braun and Clarke (2006). Analysis was conducted using NVivo, a software program that assists with coding and organizing qualitative data. The themes arising from analysis were discussed with the second author who supervised this research.

Demographic data are reported in Table 1. Fifteen adults (12 females) aged between 20 and 62 years were interviewed. Three had bilateral microtia. Three participants also had hemifacial microsomia, a congenital condition which can include a spectrum of symptoms, including microtia. Seven participants had received reconstructive surgery for their microtia, and 3 an ear prosthesis. Telephone interviews were conducted with 10 participants, 4 participated in face-to-face interviews, and 1 via Skype instant messaging. Interviews lasted between 31 and 75 minutes.

**Table 1.** Participant Demographics.

Pseudonym	Age	Gender	Unilateral/bilateral microtia	Reconstructive surgery/prosthesis	Associated condition	Hearing loss	Relationship status	Mode of interview
Maisey	20	Female	Unilateral	Surgery at 12	No	Yes	Single	Telephone
Claire	32	Female	Unilateral	Surgery at 8	No	Yes	Married	Telephone
Caroline	32	Female	Unilateral	Surgery at 14	Hemifacial microsomia	Yes	In a relationship	Face-to-face
Katy	32	Female	Unilateral	Surgery at 13	Hemifacial microsomia	No	Single	Face-to-face
Jake	35	Male	Unilateral	Prosthesis at 11	No	No	Married	Telephone
Georgina	39	Female	Unilateral	Prosthesis at 16	No	Yes	Married	Telephone
Beth	43	Female	Bilateral	Surgery at 38	No	Yes	Single	Telephone
Lesley	57	Female	Unilateral	Surgery at 26	No	Yes	Married	Face-to-face
Naomi	60	Female	Unilateral	Surgery at 47	No	Yes	Married	Telephone
Barbara	62	Female	Unilateral	Prosthesis at 60	No	Yes	Married	Telephone
Otis	23	Male	Bilateral	No	No	Yes	Single	Face-to-face
Helen	24	Female	Bilateral	No	No	Yes	In a relationship	Skype instant messaging
Charles	46	Male	Unilateral	No	No	Yes	Married	Telephone
Celine	54	Female	Unilateral	No	Hemifacial microsomia	Yes	Married	Telephone
Liz	58	Female	Unilateral	No	No	Yes	Unknown	Telephone

## Results

Three main themes were identified in the data: (1) microtia as an invisible difference, (2) surgery as a welcome opportunity, and (3) living well with microtia. Some of these themes had related subthemes.

### Theme 1: Microtia as an Invisible Difference

Microtia was inferred to be an invisible difference by participants because ears can be concealed with hair or accessories such as headbands or hats. Many spoke of their concerted efforts to hide their ears throughout their life, even if they had undergone surgery:

I never have my hair up. If I do only the bottom half of my ear will show or I’ll have two plaits or something. Like, I never just put my hair up with my ear showing. (Katy, 32, unilateral microtia, hemifacial microsomia, reconstruction)

As a consequence of participants hiding their ears, some reported that friends, colleagues, and even close family members did not know about their microtia. A couple of participants reflected that it had always been difficult to know if and when to tell people about it:

Because it was a thing that wasn’t obvious, that was hidden…as hard as it is for people that have got obvious facial disfigurements, it’s out there, it’s there straightaway for everybody to see but because this was something that would only be seen if the wind was blowing or you were swimming…you weren’t going to as soon as you meet people say “Oh look at this!” (Barbara, 62, unilateral microtia, prosthesis)

Anxiety over the possibility that their ears would be revealed was pertinent for some of the participants, particularly in public:

I would wear my hair long for hiding sake…I remember an incident just off the top of my head like, when [baby name] was in his pram, so I’m pushing him down the street, really windy and hair’s blowing out my face and it’s like “Oh my gosh, Oh my gosh, I can’t hide it!” You feel exposed and it’s terrifying almost. (Liz, 58, unilateral microtia, no reconstruction)

Some female participants reflected that males might struggle more to deal with their microtia as it is less acceptable in society for men to cover their ears with long hair:

This is the advantage of a female with microtia is that people only need to know about it if we tell them, because we can cover it up with our hair, so I do feel for males who have short hair that don’t have the option to decide whether they want to disclose it to the world or not. (Georgina, 39, unilateral microtia, prosthesis)

Most participants decided to conceal their ears as an adult (whether or not they chose reconstruction or prosthesis), however some did not:

So my defense mechanism was having long hair…it was like just covering my ears and that was a very intentional decision…it just kind of got shorter and shorter and then shaved it off in the beginning of 2017. But, yeah, that was kind of how I dealt with it…people wouldn’t see it, so they wouldn’t ask about it, so it just kind of felt more comfortable. It allowed me to go on my journey and then show it when I was happy enough…I guess it worked out. (Otis, 23, bilateral microtia, no reconstruction).

Three female participants spoke about anxiety around visits to the hairdresser due to their ear being touched or worrying that hairdressers would notice the appearance and feel of the reconstructed ear as different:

When I go to the hairdressers I’m aware of it because it feels very different, it’s harder and doesn’t…it just doesn’t feel like an ear a…I feel uncomfortable for the person who’s washing my hair. I think “they must wonder what that is”…and that’s one of those times where think “It makes so much more sense to just only have one ear”…I often think “Should I just explain?,” but I never do it because I’m not really brave enough. (Caroline, 32, unilateral microtia, hemifacial microsomia, reconstruction)

Two aspects of participants’ lives in which the (in)visibility of microtia was a particular issue were romantic relationships and employment. Some reported successfully hiding their microtia from new romantic partners for significant periods of time and not knowing when and how to disclose their condition. Adults spoke of their anxiety around becoming physically intimate due to their fear that their microtia would be “discovered.” When participants did disclose, it was often because they had come to trust their new partner with this information:

I was 32 when I got married, and none of my boyfriends up to that point knew about my ear…I think it took me a long time to meet somebody that I could trust with that information…it’s not the thing you’re going to bring up in the first two or three dates…if you’re on a date and they start to touch your hair, or stroke your neck, or whatever, I used to get very tense and I used to have to move their hand away or make an excuse. (Barbara, 62, unilateral microtia, prosthesis)

A number of participants spoke about their employment experiences in relation to their microtia. Some chose not to disclose their microtia (or any associated hearing loss) to their employer. Reasons for this included worry over potential discrimination or not considering microtia, or any hearing loss, to be a disability that would negatively impact their work:

I realized that what I’d done is effectively made it an invisible impairment…to avoid sort of possible prejudicial treatment that a lot of people with disabilities get in the workplace, but also probably [due to] a sense of being patronized. (Charles, 46, unilateral microtia, no reconstruction)

When a negative impact of microtia on employment was mentioned, this was primarily attributed to hearing loss, rather than appearance. Some felt their condition was often forgotten, perhaps because it can be concealed. Two participants spoke of their frustration about the lack of understanding employers had about the impact microtia could have on their work life and reported making their own adjustments to their work environment, rather than receiving support:

So everything in the office was the wrong way round for me, I have to have my computer on my deaf side so I can have my clients on my right side so I can hear them…getting these things put in place was torture and when I said to them “You know that I’m hard of hearing?” they still couldn’t understand why I couldn’t just keep swinging my chair round…I had to raise a grievance because nobody was listening. (Naomi, 60, unilateral microtia, reconstruction)

As I got older I did notice that in the working environment people weren’t always that understanding of the hearing side of things. I think maybe because I do cope quite well, they just forget. (Claire, 32, unilateral microtia, reconstruction)

### Theme 2: Surgery as a Welcome Opportunity

This theme reflects participants’ recollections about the decision-making process for surgical reconstruction or receiving a prosthetic ear, in addition to their experiences of the operation(s), adjusting to their new appearance, and reflections on their decision.

#### Decision-making

Most participants were aware of their diagnosis during childhood; however, a few noted learning about their diagnosis and treatment options in adulthood. Since surgical options have significantly advanced in recent years, they had reached their adulthood without knowing about ear reconstruction or prosthesis. Discovering they had microtia meant reconstruction became an option for some. Some reported that making the decision to undergo ear reconstruction was straightforward, and others more difficult:

I’ve spent my whole life desperate for something, you know…I thought “Do you know what, if I don’t go through with it I will spend the rest of my life wondering.” And it was a big decision because at 38 years old, being a bit older, having a child, and working all around that, you know? (Beth, 43, bilateral microtia, reconstruction)

Because hair needs to be kept away from the ears as part of the healing process, 2 participants spoke about the implications this had on their own decision-making:

If the reason you want it [reconstruction] done is because you don’t like the attention that’s drawn toward it [ears/microtia], it brings so much more attention to you when you have the surgery because people are aware that something’s gone wrong and you actually have to talk about it a lot more. So you kind of have to resolve this thing with yourself before you have the surgery, if that makes sense. (Caroline, 32, unilateral microtia, hemifacial microsomia, reconstruction)

I wouldn’t be able to hide the fact that I only have one ear while the surgeries are ongoing…I’m quite wary of my kids being teased, or people who don’t know about my ear suddenly knowing about my ear when they don’t need to. (Jake, 35, unilateral microtia, prosthesis)

Participants reported being happy with their decision not to undergo surgery but expressed concerns around parents making decisions about surgery on behalf of their child or that the possibility of living well with microtia was not discussed.

not to trivialise…it is JUST a little ear. It’s not a huge deformity and a huge disability, I think that it’s become “We’re going to reconstruct, we’re going to do this, we can do that”…the parents are very defensive really toward the idea that reconstruction really must be the child’s choice. (Celine, 54, unilateral microtia, hemifacial microsomia, no reconstructio

Growing up, I was offered the reconstruction, but there was no other kind of option to explore alternatives, like learning to live with it with no reconstruction. I think in hindsight it would have been good perhaps to talk to someone about my reasons for wanting it done. (Claire, 32, unilateral microtia, reconstruction)

#### Adjusting after surgery

Some participants reported adjusting well having their ear reconstructed and noticed the psychosocial benefits straight away:

I will spend the rest of my life just being in amazement by what they have done, you know? How that changes…just the relaxation and the freedom you feel. I feel inner freedom, that’s how I can explain it. You’re not carrying this…all this, I don’t know, disappointment in yourself or trying to hide all the time. (Beth, 43, bilateral microtia, reconstruction)

Participants expressed various motivations for choosing to undergo surgery. Some were intrapersonal, such as longing for 2 identical ears and the development of self-confidence that came with this. Other motivations were more behavior-based such as changing their hairstyle or wearing earrings or glasses:

Fundamentally, I just wanted two ears…one of them [friend] lost all her hair and I remember thinking “My God, if that ever happened to me, I’d not only have no hair, but everybody would know I didn’t have a proper ear.” And that was probably when I really started to really wish I’d got 2 ears. I wanted to wear earrings—it sounds silly doesn’t it? (Naomi, 60, unilateral microtia, reconstruction)

No interviewees reported regretting their decision to undergo reconstructive surgery. Some found adjusting to their new appearance challenging or described unmet expectations about the postsurgical appearance of their ears. The majority spoke about continuing to conceal their ears, suggesting they may remain conscious of their appearance and expressed an awareness that surgery was not a guaranteed solution for existing confidence issues:

You’ve literally grown up most of your life never really feeling confident in yourself, you can’t just switch that off, that doesn’t just go away, you know? That’s something that has to be worked on…it’s like becoming a new person almost. (Liz, 58, unilateral microtia, no reconstruction)

My parents pushed for it [prosthesis] and they told me it would fix all my problems…but it didn’t help with confidence. It still felt I was different to everybody else…now, as an adult in my mid 30s, if I was given the choice over again I probably would have kept my smaller ear or went for the reconstruction because I’ve developed my own confidence and accepted myself as I’ve got older, but certainly I struggled a lot when I was younger. (Jake, 35, unilateral microtia, prosthesis)

Naomi reflected on her experience of surgery, and how psychosocial support before and/or after surgery could have been beneficial:

Nothing in the wide world prepared me for what I would look like when they removed my bandaging. When the doctor removed my bandaging, he left me on my own to look in the mirror, I had nobody in that room with me. And, of course, it looks like a big rugby player’s cauliflower ear, it was colossal! I didn’t say anything to anybody because I’m not a complaining person, but that’s where I think counseling would be helpful, or support. To all intents and purposes every doctor, registrar, consultant, nurse, they were mind-blowing. It was just that after the post-op stuff that was missing. (Naomi, 60, unilateral microtia, reconstruction)

### Theme 3: Living Well With Microtia

While acknowledging the challenges, most participants spoke positively about microtia and reflected on how they had coped well with it. In doing so, they emphasized that their ear did not define them as a person. Support from family and friends featured prominently in these accounts, especially when they were not made to feel any different from other family members:

I was brought up to accept my ear and by that I don’t mean that I was made to accept it…I’m from a large family—a lot of sisters, and I was never treated any differently to any of them. If their hair was put up and plaited my hair was up and plaited so everybody could see my ear. And I think that was the right thing to do. (Celine, 54, unilateral microtia, hemifacial microsomia, no reconstruction)

You’re not going to be like “Oh my God, I love my ears,” but you will get there eventually…just surround yourself with good friends. Best friends help a lot, you know? Sometimes that’s a bit overlooked. (Otis, 23, bilateral microtia, no reconstruction)

Some participants had grown up without a diagnosis, assuming their ear was just atypically formed; this made discovering microtia a significant experience. Rather than receiving a formal diagnosis, many discovered microtia by chance, for example, searching on the internet for “little ears” or reading an article in the newspaper. Discovering they had microtia was beneficial as it resulted in them finding organizations where they could receive support and meet others with microtia:

I was 38 when I first found out “Oh this is a condition and other people have it!”…that moment of clarity, just knowing that other people have this, and then looking on the internet and getting into group and listening to people talk about it, like other adults and thinking “Oh, I am so not alone here.” (Beth, 43, bilateral microtia, reconstruction)

I went and did one of the [Microtia UK] family days, and it was really nice to meet someone who had the same thing as me and who was my age, because I’ve never had that before…and that really helped. (Maisey, 20, unilateral microtia, reconstruction)

Becoming aware of microtia also enabled participants to learn about their condition and seek information. This made them more comfortable explaining their difference to others and facilitated their search for treatment, support, and advice:

Yeah and just being able to talk to someone and say “Look I am not happy with how I look and I am not happy with my ears.” I’ve had to hide that. I’ve not been allowed to talk about it and that was just…it was a breakthrough moment in my whole life, you know? (Liz, 58, unilateral microtia, no reconstruction).

Some participants reported that making downward comparisons, through appreciating that others live with conditions or health problems that could have a greater impact than microtia, had been helpful in dealing with the condition:

I remember one time, my second operation I had a cast on my head in the…obviously when they took it off, my hair was all greasy, I looked a mess, and I had to leave the hospital that day and I was crying because I looked ugly…we walked past a little baby that was hooked up to monitors and my mum was like “You’re lucky” she was like shouting “There’s nothing wrong with you.” I think that’s when I realized it’s not a massive deal. (Maisey, 20, unilateral microtia, reconstruction)

Lastly, participants reported that focusing on other aspects of their lives (eg, career and family life) had lessened the significance of microtia. Some felt this change had come with age, as appearance became less of a focus:

It was very prominent when I was a kid, you know, a very obvious thing in my life, and as I’ve got older it’s kind of receded into the background…so even though you don’t always completely forget about it, you wouldn’t necessarily want to change it perhaps because it’s contributed to who you are. (Charles, 46, unilateral microtia, no reconstruction)

## Discussion

This study specifically focused on qualitatively exploring the experiences of adults with microtia. Despite the challenges microtia had presented over the years, adults in this study did not believe it had had a lasting negative impact on their lives and reported a number of ways in which they had lived well with the condition. Similar to research with adults with other craniofacial (Eiserman, 2001; Hamlet and Harcourt, 2015) and appearance-altering conditions (Egan et al., 2011), interviewees reported that supportive friends and family had helped them to deal with the impact of microtia. Additionally, participants felt the impact of microtia had lessened over time, when other aspects of their lives (eg, career, starting a family) became more salient than their appearance. Nevertheless, these interviews have revealed aspects of living with microtia where support or advice could be beneficial and areas that warrant further research.

The offer of surgery was welcome, although deciding to have reconstruction was challenging for some who struggled with the notion of surgery for what they considered to be aesthetic reasons. Some reported not feeling sufficiently informed about the procedure and concerns about not being able to conceal their ears with their hair during the postsurgical healing process. Those who underwent surgery reported both intrapersonal (eg, increasing self-confidence) and behavioral motivations (being able to wear glasses and earrings). Most participants did not explicitly report dissatisfaction with the outcome of their surgery, yet many continued to hide their ears with their hair and expressed ongoing anxiety about revealing them. This suggests that those who undergo ear reconstruction may struggle to overcome existing confidence issues and that surgery is not necessarily a “quick fix” or remedy. The fear of being judged negatively by others is often found in individuals with visible differences, whether or not the difference can be concealed (Newell and Marks, 2000; Sharratt et al., 2018). In addition, concealing ears with hair appeared to be an ingrained coping mechanism that had been performed for many years to avoid unwanted attention and may have been hard to eradicate, despite being satisfied with reconstruction. It is possible that undergoing ear reconstruction in childhood might have prevented this. Alternatively this behavior may suggest that participants’ aesthetic expectations for their ear were unmet. While they did not explicitly report this, they may have felt uncomfortable expressing dissatisfaction with the surgery during the interview, perhaps because they felt pleased at finally receiving treatment and did not want to appear ungrateful.

These findings highlight the importance of considering each patients’ goals and motivations for ear reconstruction and for clinicians to establish whether their expectations of surgery are realistic. Engaging patients in shared decision-making requires providing them with information that meets their individual needs and understands their motivations, as well as expectations for postsurgical aesthetic and functional outcomes. Interventions which aim to support the consideration of patients’ expectations and goals within shared decision-making about surgery might be useful to develop in this context. Additionally, as concerns about appearance were reported across the lifespan, individuals with microtia may benefit from access to psychosocial interventions specific to visible difference, for example, Face IT for adults (Bessell et al., 2012) or YP Face IT for young people (Williamson et al., 2015), which are online interventions providing support, advice, and strategies for those with appearance-related distress.

In addition to struggling to adjust to their new appearance, some participants vividly recalled their experiences of seeing their reconstructed ear for the first time. This highlights the importance of exploring patient motivations for surgery in preoperative consultations, in conjunction with highlighting the possibility that their aesthetic expectations may not be met (Aspinall, 2010). In addition, emotional support at this point in the patient’s reconstructive journey should be provided.

Among those who became aware of their diagnosis at an older age, feelings of relief were recalled and how this had allowed them to become fully informed about microtia and seek treatment. This highlights the importance of timely identification of microtia at birth to allow appropriate referrals to craniofacial and hearing specialists. Furthermore, when discussing their own decision-making, some participants commented that parents can struggle with decision-making for their child’s surgery and expressed concern that some may persuade their child to have surgery. Indeed, parents have been found to play a significant role in the decision-making process for their child’s craniofacial reconstructive surgery (Bemmels et al., 2013; Kapp-Simon et al., 2015) and can express wanting surgery sooner rather than later, especially if concerned about teasing (Johns et al., 2018). Future qualitative research should investigate decision-making for microtia among parents in greater depth to help identify any areas for psychosocial support and intervention for both parents and children throughout the treatment processes.

Participants spoke of concerns about disclosing their microtia in various situations. Revealing it to romantic partners was particularly pertinent due to fears of negative judgment and reactions, mirroring findings by Sharratt et al. (2018) who explored the experiences of romantic relationships with 22 adults with a range of visible differences, including those that can often be concealed (eg, alopecia). Taken together, these findings suggest that those with microtia may benefit from guidance and support around disclosing their difference when developing romantic relationships.

While not necessarily a barrier to employment opportunities or career trajectory, microtia (in particular hearing impairment) had still presented some challenges in these areas. Some participants were unsure whether to disclose microtia to employers. If they did disclose, some reported they received little support to make the relevant adjustments and felt their condition was sometimes overlooked. The workplace poses challenges for those with hearing loss (Southall et al., 2011), and it is important to empower individuals to disclose microtia to employers, if they wish. The development of targeted educational materials could help employers better understand microtia and its potential impact on working life, which could improve workplace experiences for those with the condition.

## Limitations

This research has provided valuable insights into living with microtia in adulthood; however, its limitations must be acknowledged. First, participants reported living well with microtia and were engaged with the charity Microtia UK. Those who came forward may have different attitudes and experiences than individuals who do not have this source of support or chose not to come forward. Therefore, we cannot assume that everyone becomes comfortable with their microtia over time. In addition, this sample comprised mostly of females, meaning the male perspective was underrepresented, despite microtia being 20% to 40% more prevalent in males (Luquetti et al., 2012). Future studies should consider how to reach and engage more men in visible difference research, as they are often underrepresented (eg, Egan et al., 2011; Sharratt et al., 2018; Stock et al., 2019; Zucchelli et al., 2020).

Furthermore, participants self-identified as having microtia, and this was not confirmed by a medical provider. In addition, this study was focused on experiences of adults in the United Kingdom, and therefore, their experiences of care may be different to other countries. Three participants also reported having hemifacial microsomia, which is a visible facial difference. This condition could have different psychosocial consequences and might have influenced the experiences reported. Lastly, participants’ ages spanned 42 years, so although they all had experience of being an adult with microtia, they grew up in different eras, and as a consequence, their treatment options and experiences varied. There is also a possibility that time affected the recall of experiences. As a result of these limitations, it is important not to generalize these results to all adults with microtia.

## Conclusion

This qualitative study focused on the psychological impact of microtia in adulthood and identified areas for support and future research. Participants had incorporated microtia into their self-concept and did not report a lasting negative impact on their lives, yet psychosocial challenges as a consequence of the condition were reported. These included anxiety around revealing their ears (even after reconstruction) and disclosing their condition to romantic partners, decision-making about reconstructive surgery, and feeling unsupported in the working environment. Individuals with microtia may benefit from psychosocial interventions to increase confidence, access to support for treatment decision-making, and support and guidance around disclosing microtia to employers.

## Appendix A

I am going to begin with some general questions that I would like you to answer if you feel comfortable.

1. How old are you?
2. Do you identify as male or female?
3. Do you have microtia on one or both sides?
4. Do you have any other associated conditions?
5. Do you have hearing loss?
6. Have you had surgery for microtia? If yes, when?
7. What is your relationship status

Now I want to learn more about your experiences of living with microtia. Remember you do not have to answer a question if you do not want to, and you can stop the interview at any time.

1. Can you tell me what life has been like over the years living with microtia?
2. What were your experiences as a child?
3. Have there been any memorable lows/highs in relation to your microtia?
4. How did you make a decision on whether to have surgery for your microtia or not? Who decided? What age were you?
5. How have you managed the difficult things in relation to your microtia throughout your life?
6. What factors do you think have helped you adjust well in living with microtia?
7. Does microtia affect your life on a regular or daily basis, if so, how?
8. Have you ever sought any information or support to help you manage your microtia?
9. What do you think of the existing support for people who have a microtia? Have you come across any?
10. Do you know anyone else who has microtia? Has (or would) that be beneficial or useful?
11. Do you think there is any information that people living with microtia need to know?
12. Is there anything else that you would like to add or share?
